# Decrease in Tongue Pressure in Frail Patients in the Sitting Position and Its Alleviation by Plantar Grounding

**DOI:** 10.3390/jcm13133697

**Published:** 2024-06-25

**Authors:** Yoshihisa Fujinami, Hideki Nosaka, Keiji Sato, Manabu Kirita

**Affiliations:** Department of Emergency Medicine, Kakogawa Central City Hospital, Hyogo 675-8611, Japan; hibo1023@hotmail.com (H.N.); ksato2501@yahoo.co.jp (K.S.); kirita.manabu@gmail.com (M.K.)

**Keywords:** tongue pressure, oral frailty, frailty

## Abstract

Purpose: The deterioration of oral function has received much attention, also being referred to as “Oral frailty”. This study evaluated the change in tongue pressure, one of the objective items of oral frailty, to examine the relationship between body position and tongue pressure. Methods: This study was a prospective, observational study conducted in a single center. The participants were categorized by their Clinical Frailty Scale (CFS) scores. Tongue pressure was measured in the following positions: dorsal, sitting, and sitting with plantar grounding. Differences in tongue pressure between CFS and between body positions were statistically analyzed. Results: A significant decrease in tongue pressure was demonstrated in CFS4 compared to CFS3. Furthermore, in CFS5 and CFS6, tongue pressure decreased in the sitting position compared to that in the dorsal position, and tongue pressure recovered to the dorsal level with plantar grounding. Conclusions: Tongue pressure decreased with the progression of frailty. It was decreased by sitting, and this decrease was alleviated by plantar grounding.

## 1. Introduction

Dysphagia is a general health problem that becomes apparent with age, even in the absence of neurological or cervical disease. At the same time, it is a serious problem that can lead to life-threatening and ADL-related health problems such as malnutrition or aspiration pneumonia. However, the risk of dysphagia cannot be assessed solely on the basis of age, as elderly people do not uniformly suffer from dysphagia [1]. Recently, the concept of frailty, as well as age, has gained importance. In addition, one of the causes of frailty is a decline in oral and swallowing functions, which is now gaining attention as oral frailty [2]. We previously reported that the deterioration of oral function was associated with the appearance of new frailty in patients admitted to the ICU, and one of the mechanisms of this association was assumed to be a secondary infection, especially aspiration pneumonia [3].

Low tongue pressure, which is one of the objective items of oral frailty [4], is associated with frailty [5] and aspiration risk [6]. Therefore, it is reasonable and beneficial for admitted patients to undergo tongue pressure measurement screening before oral intake in order to prevent aspiration. In addition, ensuring a posture that can exert stronger tongue pressure is very important in reducing the risk of aspiration. It is not yet properly recognized in clinical medicine whether changes in posture can cause instability of the head and trunk, which results in changes in oral and swallowing functions such as bite force and tongue pressure [7]. The importance of plantar grounding for trunk retention has been highlighted, especially in the sitting position; however, the importance plantar grounding has hardly been reported in global journals.

In fact, non-frail individuals do not perceive the importance of plantar grounding while sitting and can swallow regardless of their body position. We hypothesized that the importance of plantar grounding would become apparent in individuals with frailty because of the increased instability of the trunk. This study aimed to evaluate changes in tongue pressure with body position and to determine whether the progression of frailty is one of the factors contributing to these changes.

## 2. Materials and Methods

This was a single-center, prospective, observational study. A total of 67 individuals were enrolled: we enrolled 23 medical staff members as healthy controls and 44 patients admitted to the emergency department between 1 April and 31 July 2023 and treated as inpatients for at least 48 h. The enrolled patients did not receive ventilatory management, tube feeding, or central venous nutritional therapy, and all fasted for <3 days. A post-gastrostomy patient who had not taken anything orally prior to admission was excluded. Patients with vertebral or facial fractures were excluded, considering the effects on positional retention and occlusion. Patients with cognitive dysfunction were also excluded because tongue pressure measurements were unable to be performed accurately. Under these exclusion criteria, none of the patients with a Clinical Frailty Scale (CFS) score ≥ 7 were included. Thus, patients with physical and mental functions affecting tongue pressure were excluded, and frail patients who could demonstrate the same tongue pressure as before admission were selected. The diagnoses of the included patients are shown in Supplemental Table S1.

Patient background information regarding age, sex, frailty, and tongue pressure was collected. The CFS was used to assess frailty [8]. Although the CFS was updated in 2020 [9], the original CFS is still frequently used, and it has been shown to be relevant to other assessment tools for frailty [10]. Therefore, we recognized that the use of the original CFS was viable at the point of conducting the study. Tongue pressure was measured from the time of admission to the day before oral intake. Tongue pressure was measured in each of the following positions (Supplemental Figure S1): dorsal (Group D), sitting (Group S), and sitting with plantar grounding (Group SP). Tongue pressure was measured with a JM-TPM tongue depressometer (JMS, Hiroshima, Japan). To briefly explain how tongue pressure was measured, a device with a balloon-like probe was first placed in the mouth. Next, the patient was instructed to press their balloon against the probe with maximum force. The maximum pressure applied to the balloon was indicated in kPa. Measurements began with the posture the patient was in at the time of the visit; subsequent postures were determined by the patient. In the sitting position, the bed was set at a 45° gudgeon up, and a step was placed between the bed rail and the sole of the foot. Although tongue pressure and occlusal force are highly related [11] and similar changes in body position have been reported [12], we employed tongue pressure as an objective item of oral function in this study. The reason for this is that occlusal force may change depending on the number of teeth [13], considered as a major risk of bias in this study. For patients with dentures, tongue pressure was measured under the same circumstances of eating before admission.

This study was conducted in a clinical work setting, and there were few elderly patients with CFS1,2. We considered including only patients with CFS3 or higher but added healthy staff as a control group because of the advantages it would bring in terms of having a visually healthy comparison group. Therefore, a CFS1,2 group was not included in the statistical analysis.

Measurements were categorized by the CFS and presented as the mean ± standard deviation and median (interquartile range). The Mann–Whitney U test was used to compare differences in continuous variables between the groups and measurements between groups, with *p* < 0.05 indicating a significant difference. All statistical analyses were performed using GraphPad Prism software (version 7.0; GraphPad Software Inc., San Diego, CA, USA).

## 3. Results

**Figure 1:**
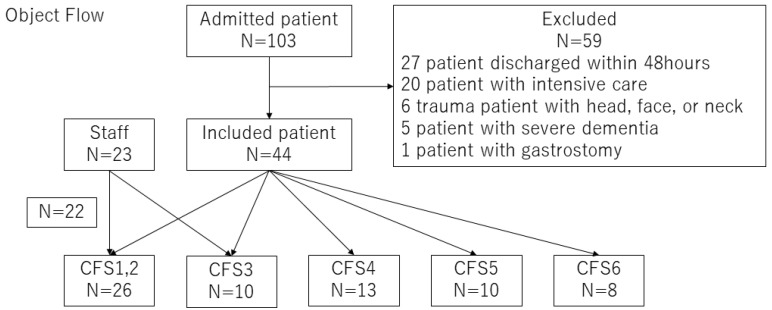
Enrolment flowchart of the study participants.

**Figure 2:**
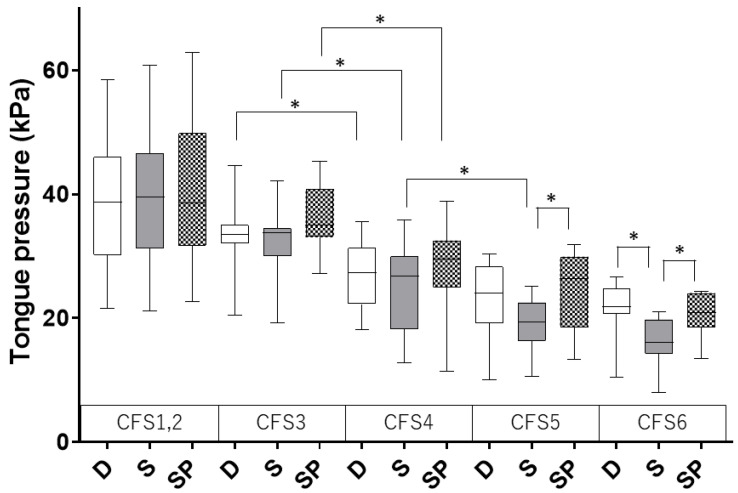
Tongue pressure per CFS and body position. CFS, Clinical Frailty Scale; D, dorsal position group; S, sitting position group; and SP, sitting with plantar grounding group. The data are expressed as the interquartile range with the boxplot. Mann-Whitney *U* test, * *p* < 0.05.

As shown in Figure 1, the participants were divided according to the CFS. Tongue pressure for each body position is shown in Table 1 and Figure 2. CFS1, 2, and 3 did not show any differences in body position. Compared to CFS3, CFS4 showed a significant decrease in all body positions (group D: 27.0 ± 5.8 vs. 33.4 ± 5.6 kPa; group S: 25.1 ± 7.9 vs. 32.5 ± 5.5 kPa; and group SP: 28.2 ± 8.1 vs. 36.3 ± 5.4 kPa; *p* < 0.05). Compared to CFS4, CFS5 showed a significant decrease only in group S (19.0 ± 4.4 vs. 25.1 ± 7.9 kPa, *p* < 0.05). No significant difference was observed between CFS5 and CFS6 (group D: 23.1 ± 5.9 vs. 21.4 ± 5.8 kPa, *p* = 0.53; group S: 19.0 ± 4.4 vs. 16.3 ± 4.9 kPa, *p* = 0.21; and group SP: 24.4 ± 6.2 vs. 20.6 ± 4.1 kPa, *p* = 0.16).

Differences in tongue pressure between body positions were observed for CFS5 and CFS6. In CFS5, there were no significant differences between group D and group S (23.1 ± 5.9 vs. 19.0 ± 4.4 kPa, *p* = 0.11); however, a significant difference was observed between group S and group SP (19.0 ± 4.4 vs. 24.4 ± 6.2 kPa, *p* < 0.05). In CFS6, group S showed a significant decrease in tongue pressure compared to group D and group SP (*p* < 0.05) (group D: 21.4 ± 5.8; group S: 16.3 ± 4.9; and group SP: 20.6 ± 4.1 kPa).

## 4. Discussion

This study was designed to determine whether the progression of frailty and postural changes affect tongue pressure in patients with frailty. In the CFS proposed by Rockwood et al. [8], Scale 4 or more is recognized as frailty. In the present study, a significant decrease in tongue pressure was observed with CFS4, and tongue pressure tended to decrease even with CFS3. Thus, decrease in tongue pressure may be a useful metric for the early recognition of the early stages of frailty. In fact, Tanaka et al. demonstrated the concept of “Oral Frailty”, which comprehensively reflects occlusal strength, swallowing function, and the bacteriological/immunological status of the oral cavity and is considered a preliminary stage of frailty [2]. In addition, we reported that oral dysfunction, represented by the loss of molar teeth, is related to the prognosis of older adults in the intensive care unit [3]. There was no difference in tongue pressure according to body position in participants with CFS4 or less. In contrast, in participants with CFS6, there was a significant decrease in tongue pressure in the sitting position (Group S) and a significant alleviation in the plantar grounding position (Group SP). Although there was no significant difference between Group D and Group S in CFS5, the degree of tongue pressure reduction in the sitting position tended to increase as frailty progressed. This mechanism can be explained by referring to reports that the instability of the head position causes a decrease in tongue pressure [7].

It is important to note that the present study examined the relationship between tongue pressure and not age but frailty. Although age and frailty are strongly correlated, they are definitely different indicators that should not be equated. In fact, it has been reported that indicators of aging such as the Aging score [14], which is calculated from physical and mental activity, decline sharply around age 60 due to a decline in physical functions such as walking speed, and again around age 80 due to a decline in mental functions such as cognitive functions [15]. Furthermore, unlike age, this score does not decline at a constant rate, suggesting that frailty is an intervening condition.

The clinical implementation of this study is not only a solution to pulmonary aspiration but also its early detection. This is because swallowing is categorized into three phases: oral, pharyngeal, and esophageal [16]. We focused on tongue pressure, which is the most important element in transmitting the mass from the oral cavity to the pharynx. Tongue pressure may vary depending on the patient’s condition and can be managed through oral care or rehabilitation. Furthermore, it can also be used as an objective indicator. Video endoscopy/video fluorography (VE/VF) is often performed in clinical practice, especially in hospitalized patients, where VE is preferred because it does not require room movement, the use of contrast media, or exposure to radiation [17]. However, VE requires highly experienced staff and is somewhat invasive to the patient. In addition to these three phases, cognitive and masticatory functions must be assessed to achieve feeding without aspiration [18,19]. Cognitive function contributes to the recognition of the size or hardness of food and movement of the appropriate amount of food into the oral cavity. Masticatory function is necessary to chew food appropriately and finely, and it concerns the number of teeth, bite strength, and salivation [20]. Cognitive function is also needed to appropriately shift to the swallowing motion. The loss of these functions can be compensated to some extent by adjusting the food form and caregiver’s ingenuity.

The interpretations of the results are as follows: The muscular strength of the oral cavity and pharynx, which are involved in tongue pressure, decreases with the progression of frailty. In addition, the finding that tongue pressure was lower in Group S than in Group D suggests that other skeletal muscles, such as the spinal muscles, compensated for the decreased tongue pressure. Furthermore, the finding that tongue pressure in Group SP was better than that in Group S suggests that plantar grounding improved the ability of skeletal muscles to stabilize the trunk in the sitting position. It makes sense that this compensatory effect would be insufficient in advanced frailty, such as CFS6, where limb muscles would be weakened.

This study has some limitations. First, the number of cases considered is small. We did not include gender differences in our analysis, citing previous reports that showed no differences in adults in their 60s and older [21]. If a multivariate analysis had been conducted with a larger number of cases and the relationship between tongue pressure and factors such as age or gender had been confirmed, the relationship between tongue pressure and frailty would have been more clearly demonstrated. However, the present study was designed to discuss changes in tongue pressure in frail patients depending on their body position. Therefore, another study is needed in the future. Second, the pressure applied to the plantar surface during plantar grounding was not measured, and the postural changes were not evaluated in detail. Although the stability of the head, neck, and trunk is important for stable chewing and swallowing [8], this mechanism was not proven in this study, and it requires further investigation in the field of kinesiology. As mentioned above, the swallowing process is so complex that it is impossible to assess swallowing function with tongue pressure alone. However, it is an important finding in clinical medicine that the progression of frailty and/or body instability causes a decrease in tongue pressure during swallowing, and we hope that avoiding oral intake in positions that decrease tongue pressure will help prevent aspiration. In this study, it was found that in frail patients, it is necessary to unify body position when assessing tongue pressure. Further studies are warranted to examine tongue pressure prior to oral intake and the incidence of aspiration pneumonia and to provide a safe cutoff value for tongue pressure for oral intake.

## 5. Conclusions

Tongue pressure showed a decreasing trend with the progression of frailty. It also decreases in the sitting position compared to the dorsal position, though this decrease is alleviated in the plantar grounding position.

## Figures and Tables

**Table 1:** Baseline characteristics of the study population.

CFS	1,2	3	4	5	6
n	26	10	13	10	8
Age (years)	32 ± 11	64 ± 7	74 ± 9	85 ± 6	85 ± 5
Sex (Male) (%)	69	80	54	40	38
Tongue Pressure (kPa)					
Dorsal position (D)	39.6 ± 10.8 40.7 [30.0, 47.2]	33.4 ± 5.6 33.6 [32.0, 35.1]	27.0 ± 5.8 27.3 [22.3, 31.4]	23.1 ± 5.9 24.1 [19.1, 28.4]	21.4 ± 5.8 21.9 [20.7, 24.9]
Sitting position (S)	40.4 ± 11.0 41.9 [30.8, 47.0]	32.5 ± 5.5 33.8 [30.0, 34.5]	25.1 ± 7.9 26.8 [18.2, 30.0]	19.0 ± 4.4 19.3 [16.3, 22.6]	16.3 ± 4.9 16.1 [14.2, 19.8]
Sitting with planter grounding (SP)	40.8 ± 11.1 39.1 [31.6, 51.0]	36.3 ± 5.4 35.0 [33.1, 40.9]	28.2 ± 8.1 29.5 [25.0, 32.6]	24.4 ± 6.2 26.4 [18.5, 29.9]	20.6 ± 4.1 20.9 [19.5, 24.1]

CFS, Clinical Frailty Scale. Data are presented as mean (standard deviation) and median (interquartile range).

## Data Availability

The datasets generated and/or analyzed during the current study are available from the corresponding author on reasonable request.

## Supplementary Materials

The following supporting information can be downloaded at: https://www.mdpi.com/article/10.3390/jcm13133697/s1, Figure S1: Illustration of each position; Table S1: Breakdown of included patients.
