# Supplementary material for: Decrease in Tongue Pressure in Frail Patients in the Sitting Position and Its Alleviation by Plantar Grounding
Source: J Clin Med. 2024 Jun 25;13(13):3697. doi: 10.3390/jcm13133697 (PMC11242899; doi:10.3390/jcm13133697)
Supplement: Supplementary file 1 [file jcm-13-03697-s001.zip › jcm-3039902-supplementary.pdf]

Supplementary Materials:

Supplemental Figure

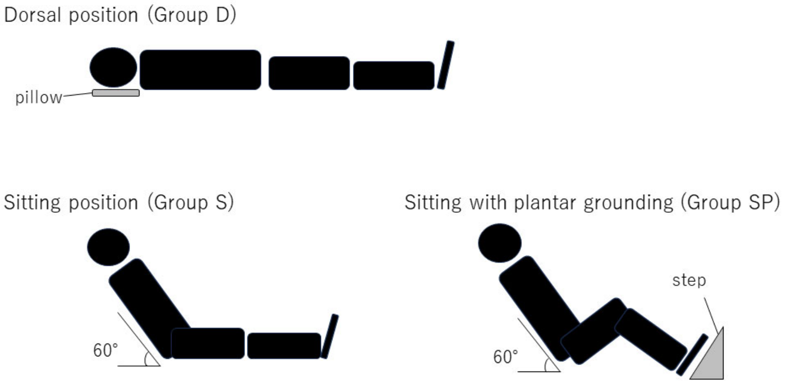

Supplementary Figure S1. Illustration of each position: pillows were placed in the supine position and the reclining angle was set at 60° in the sitting position.

Supplementary Table S1. Breakdown of included patients.

**Diagnosis**	**Number**
Urinary tract infection	12
Bone fracture (rib, upper limb)	5
Aspiration pneumonia	5
Community-acquired pneumonia	4
Hyponatremia, Hypokalemia	4
Acute drug intoxication	3
Heat stroke	3
Acute vestibular syndrome	3
Others (Bee stings, Snake bite, Dog bite)	5
